# Fracture of the Astragalus, with an Analysis of the Recorded Cases of the Injury

**Published:** 1858-10

**Authors:** Bernard Monahan


ART. III.—*Fracture of the Astragalus, with an Analysis of the Recorded Cases of this Injury.* By Bernard Monahan, M. D.*

Case I. "I. York, aged thirty-two years, being pursued by some bailiffs, jumped from the height of several feet to avoid them. The tibia and a part of the astragalus protruded at the inner ankle. I immediately returned the parts into their natural situation. Suppuration ensued, and in

---

* This paper was an inaugural Thesis presented to the Faculty of the Buffalo Medical College, 1858, and recommended by the Faculty for publication.—Ed.

five weeks a portion of the astragalus separated, and another piece a week afterwards, which, when joined, formed the ball of that bone. For three months the joint was filled with granulations; it soon afterwards healed, and the man recovered with a good use of the limb."—*Astley Cooper on Dislocations and Fractures, p.* 259, 2*d Amer. Ed.*

CASE II. *Complicated with Fracture of the Femur, &c.*

"On June 21st, 1792, Mr. Tolson, aged forty years, was thrown from a curricle, on Gerard's-cross Commons. The injury he received consisted in a compound dislocation of the tibia and fibula at the outer ankle of the left leg, with a fracture of the astragalus (the superior half of which was attached to the dislocated bones of the leg), and likewise simple fracture of the os femoris on the same side. He was conveyed to a friend's house on the common, where he had the advantage of an airy, healthy situation, with every kind of domestic attention. I saw him two hours after the accident, and found the bones protruding at the ankle through a very large wound, with the foot turned inwards and upwards, and the integuments beneath the wound exceedingly confined by the dislocated bones which descended nearly to the bottom of the foot. A considerable hæmorrhage had taken place, but was stopped by spontaneous contraction of the lacerated vessels.

"From such a formidable accident in so large a joint, there appeared very little probability of the patient's recovery without immediate amputation; I therefore requested that a consultation with some other surgeons might be held on the case, and expresses for this purpose were accordingly sent to Mr. Peason, surgeon in London, and to my brother, Mr. Henry Rumsey, surgeon, Chesterfield. While I was waiting for their arrival, the patient requested me to examine his thigh, when I plainly discovered an oblique fracture of the os femoris at its superior part. This additional evil appeared to me a great obstacle to an amputation. My brother, when he arrived, being of a similar opinion, I attempted to reduce the fractured and dislocated joint into its natural situation. This I found very difficult without first separating that part of the astragalus which was pendulous to the tibia, having its capsular ligament lacerated half way around the joint. This portion of the astragalus consisted of the broad smooth head by which it is articulated to the tibia; and of almost the whole of the inner and outer malleoli; and of about the upper half of the posterior cavity on its under surface, by which it is united to the os calcis; so that the bone was divided nearly horizontally, and the part left behind consisted of the lower half of the last-mentioned cavity, of the whole of the other or anterior cavity which connects it with the os calcis, and of the anterior portion or process by which it is articulated to the os naviculare; I therefore removed it without hesitation, being persuaded that if it had been practicable to reduce it into its original situation, so large and movable a portion of bone would have been a source of pain and irritation, and have rendered the cure more difficult and painful. I then divided that portion of the integuments of the foot which was confined by the protruded end of the tibia, and was thus enabled with ease to reduce it and the fibula into their proper situation. I applied some dossils of lint dipped in tincture of opium to the wound, and covered the whole with a poultice of stale beer and oatmeal. We then reduced the fractured femur, and placed the limb in a bent position, expecting that our greatest success would be in procuring a complete anchylosis, the failure of which I concluded would leave a useless foot. The under splint was a firm excavated piece of deal, of the shape of the leg and foot, with a hole opposite the ankle. Mr. Pearson arrived in the evening and approved of the preceding treatment, giving it as his opinion that it would be safer to attempt the preservation of the limb than to amputate, under such complicated circumstances. The wound was concealed as much as possible from the external air, and the cataplasm removed no oftener than the discharges rendered necessary."—*Astley Cooper*, *op. cit.*, *p.* 298.

CASE III. "In the month of September, 1797, a gentleman lodging in Duke street, Smithfield, in a fit of insanity, threw himself from a two pair of stairs window into the street, his feet first reaching the ground. He rose without help, knocked violently at the outer door of the house, and ascended the stairs without the least assistance, bolted the door after him and got into bed. He refused to open the door and it was obliged to be forced. A neighboring surgeon was sent for who, on viewing the case, proposed an immediate amputation, which was not acceded to by his friends; but Mr. Cooper and myself were requested to take charge of the case. On examination, it was found that there was a compound dislocation of the ankle-joint. The tibia was thrown on the inner side of the foot, and when the finger was passed into the wound, the astragalus was discovered to be shattered into a number of pieces. The loose and unconnected portions of bone were removed, and the tibia replaced; after which, lint dipped in oozing blood, was was wrapped around the lacerated parts, and the limb was placed on its outer side, with the knee considerably bent. The parts were ordered to be kept cool by the frequent application of evaporating lotions."—*Astley Cooper*, *op. cit.*, *p.* 259.

CASE IV. "July, 1818. A bricklayer, aged thirty-up, of slender make, but of good constitution and of sober habits, fell from a height of between thirty and forty feet, upon loose materials for building, and alighting upon his feet, received a very severe shock, attended with comatose symptoms, a fracture of the right thigh, a considerable contusion and laceration of the left ankle-joint, accompanied with a dislocation of the bones inwards, the tibia resting upon the inner side of the astragalus; a portion of the lower extremity of that bone was fractured; the fibula was broken about three inches above the malleolus externus, and the surrounding ligaments of the joint were lacerated; little difficulty was found in reducing the dislocation and in replacing the fractured bones; but in consequence of the violent injury done to the joint, a question arose on the propriety of amputation. As the man had enjoyed good health, and was of the constitution and habits least liable to the attack of inflammatory affection, I ventured to give him a chance of saving the limb. A union by the first intention of the external wound, as far as practicable, was attempted, and the limb was laid in the most convenient, yet relaxed and easy posture. Evaporating lotions were applied, and the strictest antiphlogistic system enjoined."—*Astley Cooper*, *op. cit.*, *p.* 275.

CASE V. "I was requested to see a lady, thirty-four years of age, who, on August the 9th, 1819, had, in a fit of insanity, jumped out of a two pair of stairs window, and produced a compound dislocation of the tibia and fibula at the outer ankle. At the lady's residence I met Mr. Stephens, a surgeon residing in Hunter street, Brunswick-square, who had been called immediately after the accident. As she appeared almost insensible, and Mr. Stephens feared an injury to the brain, he took away twelve ounces of blood. When he examined the ankle, he found the malleolus externus of the fibula projecting through the wound, but unbroken, the tibia dislocated and broken, and the foot very much turned inwards. He extended the foot, and thought that the bones had exactly returned into their natural situation; adhesive plaster was applied upon the wound, and its edges were nicely adjusted. She was placed in a mattress with the limb upon the heel, and with a splint on each side of the leg. For seven days she complained of little pain, and had but slight constitutional disturbance. On the day week from the accident I was requested to see her, and finding little local or constitutional irritation, I recommended that the limb should not be disturbed, and the dressings were not removed.

"On the tenth day from the accident, Mr. Stephens finding her in more pain, examined the wound and found that it had not adhered.

"On the twelfth day, a considerable discharge issued from the wound.

"On the sixteenth day, a slough had separated and exposed the bones, which appeared shattered and projecting. On this day I again saw her, and upon examining the ankle found the astragalus projecting, and a portion of it broken; and as the surrounding parts were dead, I removed the projecting bone. Introducing my finger into the wound as soon as the astragalus had been separated, the tibia was found to be shattered, and the os calcis broken into many pieces. As her pulse was 100, and small, and her strength was failing, I immediately recommended her to submit to the opers ation of amputation, to which she consented.

"On the Monday following, the stump was dressed by Mr. Stephens, and the greater part was adhering. Two of the ligatures separated on the tenth day, and the other came away on the sixteenth day.

"September 29th. The stump was healed, excepting about the size of the section of a pea, and she had no complaint remaining excepting a sore upon her back, and pain in her left foot.

"It is proper to mention that she had her spine and kidneys injured by the fall, so as to discharge urine tinged with blood, for three weeks after the accident.

"Upon examination of the amputated limb, the tibia split up from the malleolus internus to the length of three inches; the fibula was unbroken; the astragalus was broken and detached; and the os calcis was broken in several pieces.

"I have lately seen another of the same kind, in which I was obliged to amputate."—*Astley Cooper, op. cit., p.* 321.

Case VI. *Compound Fracture, with Dislocation of the Ankle-joint, successfully treated.* By James Mitchel, Surgeon, R. N., Willer.

"On my passage from New South Wales to Batavia, in the latter part of 1820, during a dreadful gale of wind, we were obliged to strike top-gallant mast, in doing so a seaman of the name of Robert Smith, aged 23, and of a phthisical habit, was precipitated from the main top to the quarter deck, and fell on his feet. The result of this fall, as may be expected, was dreadful. On examination I found a compound dislocation of both ankle-joints. The left ankle-joint was in a peculiar helpless state. There was an extensive lacerated wound of four inches in length, at the outer ankle, through which protruded the lower part of the fibula, with the major part of the astragalus attached to it, and showing the joint with lacerated tendons and muscles. The fibula of the left leg was fractured in its centre. The right ankle was dislocated inwards.

"Several of the tarsal bones were displaced. At the inner part of the right foot, under the ankle-joint, there was a small wound through which a tendon protruded, and from which there issued a considerable quantity of arterial blood. Both limbs were pale and lifeless.

"I reduced the right ankle joint as completely as circumstances would admit of, and applied spirit fomentations, &c.

"As for the left ankle-joint, it was in such a dreadfully shattered state that by the rules of surgery I was not warranted in attempting to save the limb, even on shore with every advantage of rest, accommodation and regimen, far less on board a merchant ship, where those comforts were wanting, and where the ship was rolling and agitated by a succession of dreadful storms that seemed to have no end, and that did not terminate for three weeks after the accident. Under these circumstances I proposed amputation, but he entreated me in so piteous a manner to make an experiment (to use his own words) to save it. After explaining to him the dangerous state of his limb, and the possible consequences resulting from such an accident to the constitution, I determined to accede to his request, and I was the more inclined to do so as I thought he would not survive the shock given to his frame. I then dissected away the broken astragalus attached to the fibula, and returned the latter bone into its place, judging it quite useless to return the broken astragalus into its situation, as I supposed it would act as a foreign body in the joint. After this, I dressed the wound, placed the whole in a proper posture, applied the many-tailed bandage, and placed long splints, hollowed out from the thigh downwards to the outer and inner part of the foot; secured his foot by tape to the bed, so as to obviate, as much as possible, the rolling of the ship. I then kept both feet wet with spirituous embrocations. After the system had rallied, and the blood began to circulate freely in the vessels, very alarming inflammation came on in the extremities with strong febrile symptoms, I combatted these with very large bleedings, saline laxatives, antimonials, and large opiates to relieve pain. When this inflammatory state had subsided, symptoms of gangrene came on, which I was enabled to remove by topical applications of cold water dressings, spirituous fomentations; and, internally, by bark, opium, wine, and a generous diet. With the latter he was most amply supplied throughout his cure by captain McKissock, who commanded the ship, and was exceedingly kind to him, administering to his wants from his own table, both in the way of viands and wine; and, indeed, it gives me great pleasure in paying a just tribute to the liberality and humanity of this gentleman."—*Edinburgh Medical Journal*, *vol.* xxvii, *p.* 305.

Case VII. "A stout young lad fell from a height of fifty feet, with a hod on his shoulder, and received a compound luxation of the astragalus inwards. He was immediately conveyed to the hospital above mentioned, and put under the care of Dr. Stephens. He lay trembling and agitated, but his skin was warm and his pulse good.

"The integuments and capsular ligament over the inner side of the ankle-joint were rent, and the surface of the astragalus, where it articulates with the os calcis, occupied and rather protruded from the wound. Dr. S. endeavored to push the bone into its place; but failing in this, he tried to pull out the bone, which was also impracticable. Extension was made, and as the bone could not be reduced into its proper site, it was extracted by dividing the ligaments that held it, with a scalpel, though not without considerable suffering on the part of the patient.

"Upon minute examination of the bone, the processes to which the ligamentum inter-fibulam anterin is attached, were found broken off. The limb was laid flexed on its outer side on a splint, and pillow, and the wound dressed with lint and roller. We had not detailed the after treatment, which appears to have been judicious; there was ultimately some flexibility of the ankle-joint, with a little deficiency in the length of the limb."—*New York Med. and Phys. Journal. Copied into the Medico-Chirurgical Review, vol.* vii., *New Series, p.* 757.

CASE VIII. *Dislocation and Fracture of the Astragalus.* With Remarks by GEORGE W. NORRIS, M. D. (Journal of Med. Sci., vol. xx, p. 378.)

"William Summerill, ostler, aged 30, was admitted into the Pennsylvania Hospital, September 26, 1831, and came under my immediate care under the direction of Dr. J. R. Barton.

"An hour previous to admission, while descending a ladder, he slipped and fell in such a manner as to throw the entire weight of his body upon the outer part of his left foot. Upon examination, the foot was found to be turned inwards and nearly immovable. A slight impression existed immediately below the lower end of the tibia, and there was a considerable hard and rounded projection on the outer part of the foot, a little below and in front of the extremity of the fibula. The skin covering this projection was reddened, but not excoriated. There was no fracture of either bones of the leg.

"These appearances rendered evident that the injury was a dislocation outwards and forward of the astragalus; and a short time after admission efforts were made by Dr. Barton, to reduce it.

"This was done after relaxing in as great a degree as possible, muscles of the leg, by fixing the knee and having assistants to keep up extension, by seizing the heel and front part of the foot; same time the bone being pushed inwards and towards the joint by the surgeon. These efforts were continued for a considerable time, but had no effect in changing the position of the bone.

"Six hours afterwards, Drs. Huston and Harris saw the patient in consultation, when efforts were again made at reduction, which not proving more effectual than in the first trial, the excision of the joint was determined on.

"The patient being properly placed, an incision was made through the integuments, parallel with the course of the tendons, commencing a short distance above the projection on the foot, and extending down far enough to expose fairly the astragalous and its torn ligaments. The bone was then seized with a forceps and easily removed after the division of a few ligamentous fibres, that continued to connect it to the adjoining parts.

"Very little hæmorrhage occurred, two several vessels only requiring the ligature.

"After removal, it was discovered that about one-half of the surface which play in the lower end of the tibia, had been fractured, and remained firmly attached to the extremity of that bone, and as it was judged that the efforts to remove it would be likely to produce more injury to the joint, than would arise from allowing it to remain, no attempt was made to extract it.

"The joint being carefully sponged out, the sides of the incision were brought accurately together by means of sutures and adhesive straps, after which simple dressings and a roller were applied, and the foot restored to its natural situation, and placed in a fracture box."

Case IX. *Compound inward Dislocation of the lower end of the Tibia, with Fracture of the Tarsal Bones.*

"Elizabeth Sales, a German immigrant, aged 78 years. Accident occurred from being knocked down and crushed when passing under a canal bridge. I saw her three weeks after the injury was received, in consultation with Dr. Sprague. Over the inside of the ankle there existed a large wound communicating with the joint, exposing the astragalus and calcanium, both of which bones were broken. The patient had been exceedingly feeble since the accident, and Dr. Sprague had not judged it proper to amputate. The bones remained unreduced, but they were as comfortable as possible with pillows. Dr. Sprague had never hoped to save the limb, but he had only waited a favorable opportunity to make the amputation, which did not seem yet to have arrived.

"She died two weeks from the day which I saw her, and about four weeks from the receipt of the injury."—*Report on Dislocations with especial reference to their Results. By Frank H. Hamilton, of Buffalo. Transactions of the State Medical Society of the State of New York*, 1855, *Case* XVI, *p.* 84.

Case X. "The astragalus may be broken singly without any of the other bones being injured, by the tibia being forcibly thrust against it, as when a person jumps from a height, and comes to the ground with the tibia placed vertically on the astragalus. The shock may be sufficient to split the bone. I once saw a case of the kind produced in the above manner; there were no symptoms to indicate the nature of the accident, as there appeared to be only swelling of the joint. The patient was treated for a severe sprain, there being reason, however, to suspect a fracture. The inflammation of the joint became so great, and the man's constitution so much affected, that he died on the twelfth day. The case was considered peculiar from the severity of the symptoms. On opening the joint, however, after death, the astragalus was found to be split in two or three directions, which fully accounted for the constitutional symptoms and the other serious effects produced by it."—*Amesbury on Fractures.*

*Remarks.* Fracture of the astragalus is of very rare occurrence; of 461 cases of fracture of different bones, seen by Dr. Hamilton, only one was a fracture of the astragalus. Fracture of this bone almost always occurs, judging from the few cases that we find on record, as the result of the two following accidents: either a fall from a height, or a general crush of the bones of the foot from external violence, as when some heavy object falls upon or passes over them, such as a loaded wagon, or a railroad car. It occurs more frequently from the former accident than the latter, that is, from falls from a height. Of the ten cases of fracture of this bone, which I have collected, nine were caused by falls from a height, while only one was caused by a general crush of the bones of the foot. This latter example occurred in the practice of Dr. Hamilton, and is reported in his article on Dislocations, in the Transactions of the State Medical Society for 1855, Case XVI, p. 87.

Malgaigne, in his Treatise on Fractures, when speaking of the astragalus, says, that in a general crushing of the bones of the foot, when all the other bones are crushed or fractured, the astragalus generally escapes unharmed. How far this is true, is perhaps a question to be determined by future observations. Of simple fracture of the astragalus not complicated with fracture of the other bones of the tarsus, we have found recorded two cases, one of which was not recognized as such during life. The patient was treated for a severe sprain. The injury done to the joint was accompanied with such an intense degree of inflammation, that the man died on the twelfth day of its occurrence. In the other case, the astragalus was displaced to the outer ankle, a little below and in front of the extremity of the fibula. The skin covering this projection was reddened, but not excoriated. An incision was made parallel to the tendons through which the fractured bone was extracted.

Fracture of the astragalus is almost always compound, and accompanied with dislocation of one or more bones of the leg, thigh, or foot. Case I, was complicated with a compound dislocation of the tibia. Case II, with a compound dislocation of the tibia and fibula, and also complicated with a simple fracture of the femur. Case III, was a compound fracture of the astragalus, complicated with a dislocation of the tibia inwards. The astragalus was broken into a number of pieces. Case IV, complicated with compound inward dislocation of the tibia, fracture of the femur, and also fracture of the fibula, three inches above the malleolus externus. Case V, the tibia was found split from the malleolus internus to the extent of three inches, the fibula being unbroken; the astragalus was broken and detached, and the os calcis broken into a number of fragments. Case VI, complicated with compound dislocation of the ankle-joint, and fracture of the fibula on the same side through its centre. Case VII, complicated with compound dislocation of the ankle-joint. Case VIII, simple fracture of the astragalus with the fragments displaced outwards. Case IX, complicated with fracture of the os calcis. Case X, simple fracture not recognized as such during the lifetime of the patient.

We see by the foregoing analysis, that two were complicated with fracture of the femur through its middle: both cases occurring on the same side with the broken astragalus. Two were complicated with the os calcis. One was complicated with fracture of the tibia; two with fracture of the fibula; one occurring three inches above the malleolus externus, and the other in the centre of that bone.

Fracture of the astragalus may occur in almost any direction; it may happen antero-posteriorly, laterally, or horizontally. We find only two cases in which the direction of the fracture was described. In Case I, it occurred anterio-posteriorly, and also horizontally, breaking off the articulating portion of the bone, which joins the tibia and fibula, and also dividing it into two portions, which, when joined, formed the ball of that bone. The other example, Case II, was fractured nearly horizontally; the part left behind consisted of the portion which connects with the os calcis, and the anterior portion consisted of the part by which it is connected with the os naviculare.

The diagnosis of fracture of the astragalus is always easily made, when it is connected with an external wound communicating with the joint. But in cases of simple fracture I cannot imagine how we can determine its existence positively, unless the fragments are displaced. In such a case we might possibly make out the diagnosis; yet it would be extremely difficult, owing to the swelling and laceration, which would necessarily result from such an injury done to the joint. In case we are in doubt, we should treat it as a case of severe sprain, for probabilities are greatly against the occurrence of such extraordinary accidents, to which class these injuries certainly belong.

The treatment in accidents of this character when associated with an external wound, and where the bone is shattered and displaced from its natural situation, consists in extracting the loose and shattered fragments, by dividing any remaining attachments which may exist, with a scalpel. As a general rule, this is not followed by any considerable amount of hæmorrhage, since we rarely have to tie more than one or two small vessels. After having extracted the bone, or bones, we should sponge out the joint, bring the edges of the wound together, by means of sutures and adhesive straps, and cover the part with lint and a roller, then place the limb on its outside, on a splint, hollowed out and properly padded, having, at the same time, returned the foot to its natural situation. Evaporating lotions should be applied for some time to keep down inflammation. If symptoms of gangrene supervene, we should use warm fomentations applied frequently to the part, such as warm spirit lotions, &c. We must support the patient's strength by the administration, internally, of bark and wine, conjoined with a generous diet.

In simple fracture we can do very little except to keep the parts at rest, use cooling lotions, and combat inflammation by bleeding, saline laxatives, and antimonials.

The strictest antiphlogistic regimen should be enjoined. But when there is a simple fracture, with considerable displacement of the fragments, we should, I think, cut down to the bone, making the incision parallel with the course of the tendons, and extract the loose and displaced fragments. After which, the edges of the incision should be brought into as perfect apposition as possible, by which practice we may expect the greatest part of the wound to unite by first intention.

After extracting the astragalus, it must not be expected that the patient will recover with a perfect use of the joint, for we see by the results of the foregoing cases, that the joint usually remained stiff for a long time, a result which we should anticipate from the commencement. In such cases we have mobility commencing gradually in the joint until in the course of time the patient has a pretty good use of it. There is, in fact, a new articulating surface formed by the consolidation of the tissues in the immediate neighborhood of the joint, which comes at length to be almost as useful as before the occurrence of the injury. A slight amount of shortening of the limb, however, always remains, which can hardly be noticed in the gait, but may be shown by accurate measurement.

